# Dynamic change in the peritoneal cancer index based on CT after chemotherapy in the overall survival prediction of gastric cancer patients with peritoneal metastasis

**DOI:** 10.1007/s00432-024-05707-4

**Published:** 2024-04-30

**Authors:** Yi-Yuan Wei, Jie-Yuan Cai, Lin-Lin Wang, Jie Yang, Yan-Ling Li, Xiao-Ting Li, Xiao-Tian Zhang, Yan-Jie Shi, Lei Tang

**Affiliations:** 1 Key Laboratory of Carcinogenesis and Translational Research (Ministry of Education), Department of Radiology, Peking University Cancer Hospital and Institute, No. 52 Fucheng Road, Hai Dian District, Beijing, 100142 China; 2 Key Laboratory of Carcinogenesis and Translational Research (Ministry of Education), Department of Gastrointestinal Oncology, Peking University Cancer Hospital and Institute, No. 52 Fucheng Road, Hai Dian District, Beijing, 100142 China

**Keywords:** Peritoneal metastasis, Gastric cancer, Peritoneal cancer index, Computed tomography, Survival

## Abstract

**Purpose:**

The purpose of this research was to investigate the efficacy of the CT-based peritoneal cancer index (PCI) to predict the overall survival of patients with peritoneal metastasis in gastric cancer (GCPM) after two cycles of chemotherapy.

**Methods:**

This retrospective study registered 112 individuals with peritoneal metastasis in gastric cancer in our hospital. Abdominal and pelvic enhanced CT before and after chemotherapy was independently analyzed by two radiologists. The PCI of peritoneal metastasis in gastric cancer was evaluated according to the Sugarbaker classification, considering the size and distribution of the lesions using CT. Then we evaluated the prognostic performance of PCI based on CT, clinical characteristics, and imaging findings for survival analysis using multivariate Cox proportional hazard regression.

**Results:**

The PCI change ratio based on CT after treatment (ΔPCI), therapy lines, and change in grade of ascites were independent factors that were associated with overall survival (OS). The area under the curve (AUC) value of ΔPCI for predicting OS with 0.773 was higher than that of RECIST 1.1 with 0.661 (*P* < 0.05). Patients with ΔPCI less than −15% had significantly longer OS.

**Conclusion:**

CT analysis after chemotherapy could predict OS in patients with GCPM. The CT-PCI change ratio could contribute to the determination of an appropriate strategy for gastric cancer patients with peritoneal metastasis.

## Introduction

Peritoneal metastasis (PM) is common in metastatic gastric cancer (GC), with an estimated incidence of 55–60% (Cutsem et al. 2016; Thomassen et al. 2014; Allen et al. 2019). PM is the most frequent cause of death in patients with gastric cancer (Allen et al. 2019). Systemic chemotherapy demonstrated significantly better survival and showed promising outcomes for patients with GCPM (Thomassen et al. 2014; Guimbaud et al. 2014; Kang et al. 2012)_._ However, a cohort of patients may respond poorly to chemotherapy. For patients who could not benefit from chemotherapy, molecular targeted therapy, immune checkpoint inhibitor therapy, or intraperitoneal chemotherapy was recommended as an alternative treatment. Thus, timely distinguishing of individuals who respond poorly to chemotherapy from responders can assist clinicians in better adjusting treatment strategies as soon as possible.

Imaging modalities play a key role in assessing treatment response, including computed tomography (CT), magnetic resonance imaging (MRI), positron emission tomography (PET), and laparoscopy. Due to its fast scanning and high spatial resolution, CT is now the first choice for peritoneal imaging, and it is recommended to assess gastric cancer in the ESMO guidelines (Saiz Martínez et al. 2021). In general, an evaluation of tumor burden changes after cancer therapy based on CT is performed using Response Evaluation Criteria in Solid Tumours (RECIST) guidelines in clinical practice. However, the RECIST guidelines are limited in evaluating treatment responses for PM patients (Eisenhauer et al. 2009). PM of gastric cancer often appears to diffuse infiltration of the peritoneum, irregular shape, less than 10 mm in length, or variation in visceral distention and is not applicable for measurement under those conditions (Cobelli et al. 2013; Zheng et al. 2019).

PCI is a scoring system that helps predict long-term outcomes in PM and has been recognized as the most useful and reliable prognostic method to assess tumor plant burden in abdominal and pelvic examinations (Jacquet et al. 1993; Koh et al. 2009). PCI obtained from CT has been reported to have excellent performance in assessing tumor burden and predicting prognosis (Panagiotopoulou et al. 2021; Han et al. 2023). To the best of our knowledge, PCI change after therapy to assess treatment response and predict prognosis has not been undertaken by CT. Furthermore, changes in other radiological features and clinical characteristics after PM treatment in gastric cancer patients have not been the focus of previous reports.

Herein, this research aimed to assess the efficacy of contrast-enhanced CT scan before and after chemotherapy to predict the overall survival of PM from gastric cancer and to contribute to assisting clinicians in choosing further effective treatment therapy for PM patients.

## Methods

### Study population

**Fig. 1:**
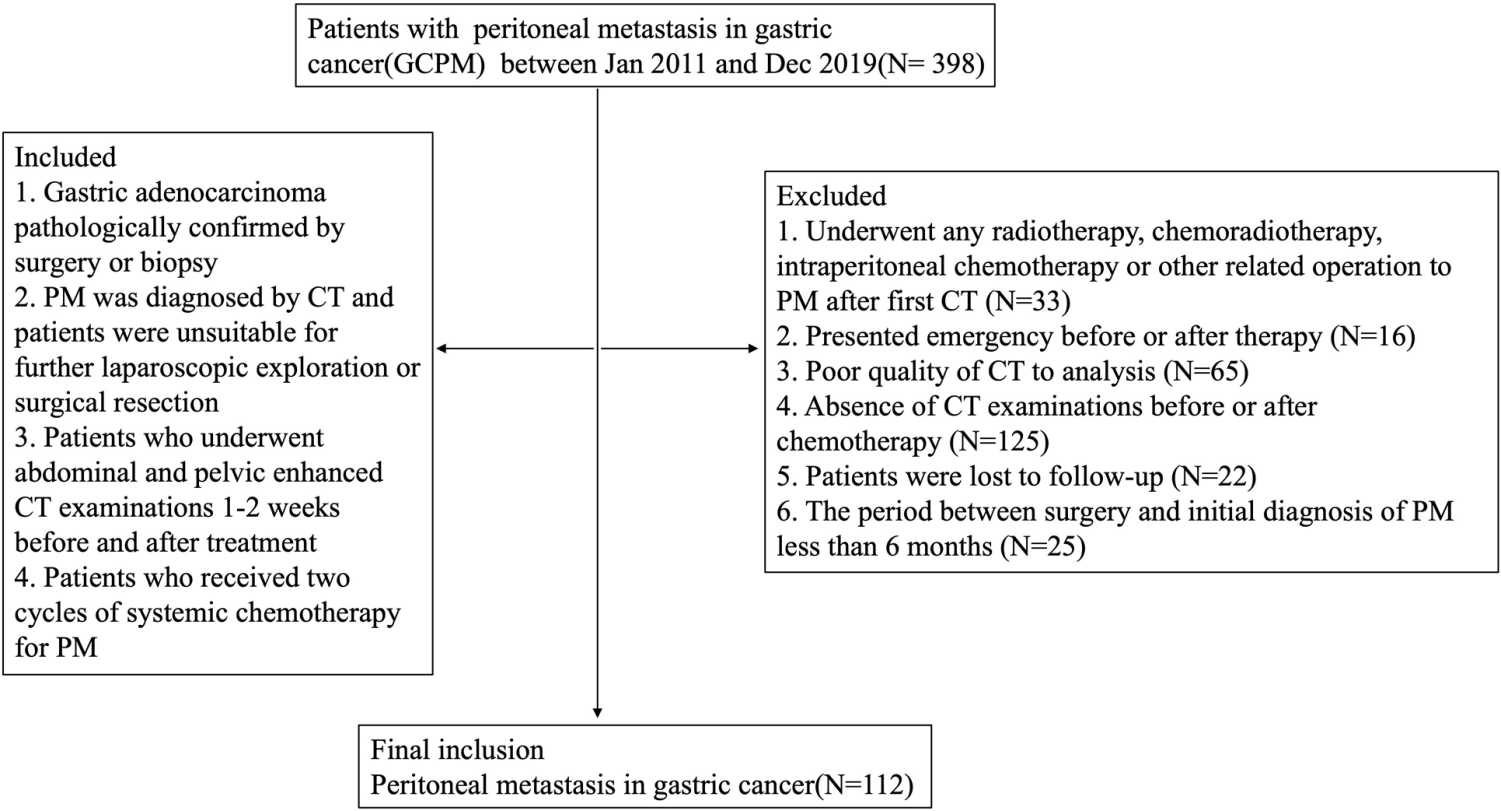
Flow chart of patient selection

A total of 398 consecutive patients with gastric cancer who were first diagnosed with PM were retrospectively enrolled in our hospital from January 2011 to December 2019. The inclusion criteria were as follows: (1) gastric adenocarcinoma was pathologically confirmed by surgery or biopsy; (2) PM was diagnosed by CT and patients were unsuitable for further laparoscopic exploration or surgical resection; (3) patients who underwent abdominal and pelvic enhanced CT examinations 1–2 weeks before and after treatment; and (4) patients who received two cycles of systemic chemotherapy for PM. The exclusion criteria were as follows: (1) poor quality of CT images (obviously evident artifacts, unable to evaluate any peritoneal regions of the abdomen and small bowel); (2) patients who presented emergency before or after therapy, including gastrointestinal perforation and bleeding; (3) patients who underwent any radiotherapy, chemoradiotherapy, intraperitoneal chemotherapy or other related operation to PM after the first contrast-enhanced CT scan; (4) absence of CT examinations before or after chemotherapy; and (5) loss of follow-up. Finally, 112 individuals who met the criteria were registered. The whole patient enrolment procedure is summarized in Fig. 1.

### CT examination

All patients fasted for 6 hours prior to contrast-enhanced CT scan. All MDCT examinations of the abdomen and pelvis before and after treatment were performed using 64-row CT (General Electrical Medical Systems, Milwaukee, WI, USA). All patients underwent plain and contrast-enhanced CT scans in a supine position. Generally, the scan began at the top of the diaphragm and extended through the plane of the symphysis pubis. The contrast-enhanced CT scan parameters were applied as follows: tube voltage of 120 kVp; mA autoregulation between 200 and 400 mA, and setting the noise index as 9; detector collimation with 64 × 1.25 mm; 0.6 s/rotation gantry rotation speed; and helical pitch of 1.5. Axial, coronal, and sagittal images with a section width of 5.0 mm were reconstructed using Advantage Workstation 4.4. The nonionic contrast medium Iohexol (Omnipaque 300; GE Healthcare) was injected at a rate of 3.0 ml/s through the median cubital vein. The dose of contrast medium was calculated according to a dose of 1.5 ml/kg. Arterial and portal phases were applied to all patients with a 35-s and 60-s delay after the start of the contrast medium injection.

### Image interpretation

All CT images before and after treatment were independently reviewed by two observers (Dr. Wei and Dr. Shi, with 7 and 13 years of experience in abdominal tumours, respectively). Observers knew that the study population was PM in gastric cancer. However, they were blinded to detailed information about clinical data and imaging results. For qualitative assessment, if there was a discrepancy, the third radiologist reviewed the images together and made a consistent evaluation of the tumor. Axial portal-enhanced images were used for measurement and assessment combined with coronal and sagittal reformations.

The distribution and size of peritoneal metastases in gastric cancer were evaluated and scored using the Sugarbaker’s PCI scoring system (Jacquet et al. 1993). The abdomen included nine regions as follows: central (No. 0), right upper (No. 1), epigastrium (No. 2), left upper (No. 3), left flank (No. 4), left lower (No. 5), pelvis (No. 6), right lower (No. 7), and right flank (No. 8). The small intestine consisted of four regions as follows: upper jejunum (No. 9), lower jejunum (No. 10), upper ileum (No. 11), and lower ileum (No. 12). The burden of PM present in each region was graded on a score of 0–3 by measuring the largest metastatic lesion. Score-0 denoted the absence of lesions, score-1 indicated lesions less than 5 mm, score-2 between 5 mm and 5 cm, and score-3 indicated lesions larger than 5 cm or a confluence. The range of the final numerical score in a patient was 0–39. The burden grade of PM was classified as low volume with total PCI scores ranging from 0 to 9, moderate volume with PCI scores ranging between 10 and 19 and high volume with PCI scores greater than 20 (Goswami et al. 2019). The change in tumor burden after therapy was ranked as reducing volume, increasing volume, and stable volume. The CT-PCI change ratio after chemotherapy (ΔPCI) was calculated as follows: ΔPCI = (PCI after chemotherapy – PCI before treatment)/PCI before treatment.

Ascites were classified as massive, moderate, mild, and none types (Nakajima et al. 2020; Honda et al. 2020). Massive ascites were considered when they extended throughout from the pelvic cavity to the upper abdominal cavity continuously, whereas mild ascites were defined as being located only in the upper or lower abdomen and pelvis. Moderate ascites indicated ascites were detected in both the upper and lower abdominal cavities. When ascites were not detected by CT scan, no ascites were recorded. The change in ascites was classified as reducing the volume of ascites, increasing the volume of ascites, and stabilizing the volume of ascites.

### Clinical evaluation

Demographic characteristics, including sex, age, and laboratory indicators (CEA, CA199, and CA 125), before and after therapy were analyzed for all patients. Gastric cancer differentiation was pathologically identified on biopsy or surgery and classified as moderate and poor differentiation. Lauren’s classification included mixed, diffuse and intestinal types. Body mass index (BMI) before PM treatment was calculated based on height and weight. Other clinical variables, including gastric cancer resection, the presence of PM at baseline and the line of therapy for PM, were also evaluated.

The response of PM in gastric cancer was assessed according to RECIST 1.1 guidelines after chemotherapy by two radiologists (Dr. Wei and Dr. Shi) (Eisenhauer et al. 2009). PM lesions less than 10 mm were considered as nontarget lesions and qualitatively assessed as complete response (CR), non-CR/non-PD, and progressive disease (PD). A PM lesion with a long dimension greater than 10 mm was considered a target lesion and the number of target lesions was ≤ 2. PD was defined as follows: (1) an increase ≥ 20% in the sum diameter of the target PM lesions; (2) the presence of new lesions; and (3) the progression in nontarget lesions. Patients with PM were finally classified into PD and non-PD groups according to RECIST 1.1 guidelines.

### Treatment strategy

The first-line regimen of systemic therapy includes chemotherapy or chemotherapy combined with immunotherapy. The chemotherapy options were XELOX (oxaliplatin 130 mg/m^2^ ivggt. day 1. Capecitabine, 1000 mg/m^2^, p.o. bid. day 1–14. Every 3 weeks) or SOX (oxaliplatin 130 mg/m^2^, ivggt. day 1. S-1, 40 mg/m^2^, day 1–14. Every 3 weeks).

### Follow-up

Patients were regularly followed after chemotherapy. Follow-up included outpatient interviews at 3 months for 2 years and then at 6-month intervals until gastric cancer-specific death. Overall survival (OS) was calculated between the first CT examination and cancer-specific death dates. At the last follow-up, patients alive or dead for other reasons were recorded. The cut-off date was December 30, 2022.

### Statistical analysis

Variables are shown as mean ± standard deviation or numbers with percentages. Univariate Cox proportional hazards models were applied to screen for prognostic factors associated with OS, and hazard ratios were calculated with 95% confidence intervals (CIs). Variables with *P* < 0.1 from the univariate analysis were substituted into the multivariate Cox proportional hazards model, and the adjusted hazard ratios were obtained. Spearman’s rho was calculated between every two variables that were significant in univariate analysis. When a correlation coefficient > 0.6 was obtained, the variable with the smaller P was chosen for multivariate analysis. The receiver operating characteristic (ROC) curve and the area under the ROC curve (AUC) with its 95% CI were used to evaluate the diagnostic performance of the predictive model for 12-month OS. Kaplan‒Meier curves with log-rank estimates were applied to compare survival curves between risk groups. Interobserver agreements were evaluated using the weighted kappa coefficient, and > 0.75, 0.40–0.75 or ≤ 0.40 were considered indicative of good, moderate or poor agreement, respectively. Subpopulation treatment effect pattern plot (STEPP) analysis was conducted to present the correlation between 12-month OS and identified prognostic factors. Pearson's r was calculated.

All statistical analyzes were performed using the Statistical Program for Social Sciences, version 25.0 (IBM Corporation, Armonk, NY, USA). A two-sided *P* < 0.05 was regarded as statistically significant.

## Results

### Patients’ clinical characteristics

A total of 112 gastric cancer patients with PM were enrolled, including both male (*n* = 71) and female (*n* = 41) patients, with a mean age of 54.67 ± 12.34 years. The clinical characteristics of the patients are shown in Table 1. In the univariate analysis, response to first-line chemotherapy was correlated with better OS than second-/later- therapy line (*P* = 0.005). Patients without liver metastases proved to have a longer OS than those with liver metastases (*P* = 0.016). The line of chemotherapy was positively associated with OS, while liver metastasis was negatively correlated with OS. The median follow-up time was 12 months and the range was from 2 to 57 months. Six patients (5.4%) were alive until the data were obtained.

**Table 1:** Clinical demographic data and univariate analysis of patients with PM in gastric cancer

Characteristics	Case (%) *N* = 112	Univariate analysis		
		*B* value	HR (95% CI)	*P* value
*Sex*				0.533
Male	71 (63.4%)		Reference	
Female	41 (36.6%)	− 0.129	0.879 (0.586–1.318)	
Age (years)	54.67 ± 12.34	− 0.013	0.987 (0.972–1.003)	0.11
BMI	21.36 ± 3.25	− 0.022	0.978 (0.920–1.04)	0.483
*Differentiation*				0.475
Well and moderate	17 (15.2%)		Reference	
Poor	95 (84.8%)	− 0.208	0.812 (0.459–1.437)	
*Treatment protocol*				0.4
Chemotherapy	81 (72.3%)		Reference	
Chemotherapy + other	31 (27.7)	0.187	1.206 (0.779, 1.866)	
Presence of PM*				0.791
Synchronous	74 (66.1%)		Reference	
Metachronous	38 (33.9%)	− 0.056	0.946 (0.626, 1.428)	
*Lauren classifications*				0.63
Mixed	29 (25.9%)		Reference	
Diffused	53 (47.3%)	− 0.196	0.822 (0.505, 1.338)	
Intestinal	30 (26.8%)	− 0.253	0.776 (0.447, 1.349)	
*History of gastrectomy*				0.667
Yes	43 (38.4%)		Reference	
No	69 (61.6%)	− 0.085	0.919 (0.616, 1.369)	
*Therapy line*				0.005
First line	86 (76.8%)		Reference	
Second- or later- line	26 (23.2%)	0.642	1.900 (1.215, 2.972)	
*Liver metastases*				0.016
No	99 (88.4%)		Reference	
Yes	13 (11.6%)	0.731	0.481 (0.266, 0.872)	
*Bone metastases*				0.796
No	108 (96.4%)		Reference	
Yes	4 (3.6%)	0.133	1.142 (0.417, 3.129)	

*B* regression coefficient, *CI* confidence interval, *BMI* body mass index, P*M* peritoneal metastasis

^*^The diagnosis of peritoneal metastasis (PM) made simultaneously with gastric cancer (GC) is referred to as synchronous GCPM, whereas metachronous GCPM refers to the emergence of PM (usually at least 6 months) after the primary diagnosis of GC. Data are presented as the mean ± standard deviation

### CT analysis for predicting long-term survival

Interobserver agreements were assessed using the weighted kappa coefficient. The kappa coefficient to assess the PCI score of each region in CT for two independent radiologists was 0.564–0.792 (Table 2), indicating good and moderate agreement. The total kappa coefficient for assessing the PCI score was 0.850, indicating a good agreement.

**Table 2:** Interobserver agreements of the two radiologists for assessing PCI scores

Regions of PCI	Kappa coefficient
PCI-0	0.731
PCI-1	0.564
PCI-2	0.633
PCI-3	0.719
PCI-4	0.621
PCI-5	0.570
PCI-6	0.616
PCI-7	0.609
PCI-8	0.627
PCI-9	0.643
PCI-10	0.673
PCI-11	0.581
PCI-12	0.792
Total	0.850

*PCI* peritoneal cancer index

The results of the univariate analysis of quantitative parameters and CT measurements for PM in gastric cancer are listed in Table 3. In laboratory examination, increasing the value of CA 125 after therapy and increasing the value of CA 199 at baseline and after therapy were the high-risk factors for OS (all *P* < 0.05). The risk of death in the group with CA 125 and CA 199 returning to normal after treatment was lower than that in the abnormal group. It revealed that lower CT-PCI at baseline and after therapy was associated with longer overall survival (*P* < 0.05). Lower burden grade of CT-PCI in baseline and after therapy was correlated with a decreased risk of OS (*P* < 0.05).

**Table 3:** Univariate analysis of quantitative parameters and CT measurements for patients with PM in gastric cancer

Parameters	Mean value	Univariate analysis		
		*B* value	HR (95% CI)	*P* value
*CA 125 at baseline*				0.124
Normal	35 (31.2%)		Reference	
Increasing	77 (68.8%)	0.336	0.715 (0.466, 1.096)	
*CA 125 after therapy*				0.001
Normal	68 (60.7%)		Reference	
Increasing	44 (39.3%)	0.703	2.02 (1.355, 3.011)	
*CA 199 at baseline*				0.024
Normal	67 (59.8%)		Reference	
Increasing	45 (40.2%)	0.464	1.591 (1.064, 2.378)	
*CA 199 after therapy*				0.002
Normal	73 (65.2%)		Reference	
Increasing	39 (34.8%)	0.646	1.908 (1.256, 2.897)	
*CEA at baseline*				0.27
Normal	61 (54.5%)		Reference	
Increasing	51 (45.5%)	0.256	1.292 (0.872, 1.915)	
*CEA after therapy*				0.179
Normal	69 (61.6%)		Reference	
Increasing	43 (38.4%)	0.27	1.31 (0.883, 1.944)	
CT-PCI at baseline	17.13 ± 8.39	0.026	1.026 (1.003, 1.05)	0.026
*CT-PCI Burden grade at baseline*				0.027
Low/moderate	71 (63.4%)		Reference	
high	41 (36.6%)	0.298	1.347 (1.034, 1.753)	
CT-PCI after therapy	15.12 ± 8.48	0.084	1.088 (1.059, 1.118)	< 0.001
*CT-PCI Burden grade after therapy*				< 0.001
Low/moderate	74 (66.1%)		Reference	
High	38 (33.9%)	0.66	1.936 (1.492, 2.511)	
Change of CT-PCI	− 2.05 ± 6.31	0.088	1.092 (1.051, 1.135)	< 0.001
Δ PCI (%)	− 9.31 ± 40.73	1.504	4.502 (2.738, 7.402)	< 0.001
*CT-PCI burden change after therapy*				< 0.001
Reducing or stable	99 (88.4%)		Reference	
Increasing	13 (11.6%)	1.318	3.737 (1.965, 7.309)	
*Ascites grade at baseline*				0.23
None/mild	50 (44.6%)		Reference	
Moderate/massive	62 (55.4%)	0.129	1.137 (0.922, 1.403)	
*Ascites grade after therapy*				< 0.001
None/mild	68 (60.7%)		Reference	
Moderate/massive	44 (39.3%)	0.625	1.867 (1.484–2.35)	
*Ascites grade change*				< 0.001
Reducing or stable	68 (60.7%)		Reference	
Increasing	44 (39.3%)	1.852	6.37 (3.522, 11.519)	
*RECIST 1.1 evaluation*				< 0.001
Non-PD	89 (79.5%)		Reference	
PD	23 (20.5%)	1.395	4.034 (2.407, 6.762)	1.395

Data are presented as the mean ± standard deviation

*CA* cancer antigen, *CEA* carcinoembryonic antigen, *CT* computed tomography, *PCI* peritoneal cancer index, ∆PCI *PCI* change ratio based on CT after treatment, *PD* progressive disease

**Fig. 2:**
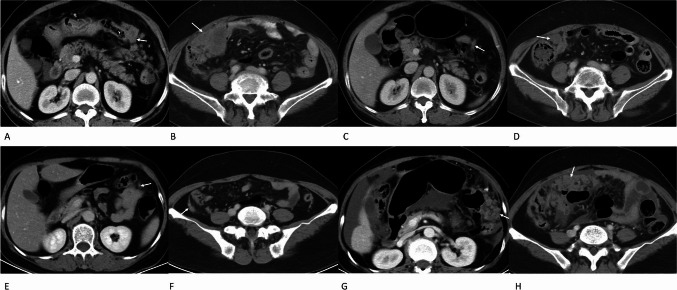
A 61-year-old woman with PM in gastric cancer (**A**, **B**). Enhanced CT images before chemotherapy showed confluent PM lesions larger than 5 cm in the left upper region of No. 3 (**A**) and the right lower region of No. 7 (**B**), indicating a score of 3, respectively. After first-line chemotherapy, the lesions decreased in No. 3 with a score of 2 (**C**) and No. 7 with a score of 2 (**D**). The total CT-PCI score was 18 after chemotherapy compared with the PCI score of 30 in the baseline CT. The OS of this patient was 13 months. A 56-year-old woman with PM in gastric cancer (**E**–**H**). Enhanced CT images before chemotherapy showed a PM nodule less than 5 mm in the left upper region of No. 3 with a score of 1 (**E**) and a 10 mm PM nodule in the right lower region of No. 7 with a score of 2 (**F**). After first-line chemotherapy, the lesions increased in No. 3 with a score of 3 (G) and No. 7 with a score of 3 (**H**). The total CT-PCI score increased to 33 after first-line chemotherapy from a PCI score of 21 on baseline CT. The OS of this patient was 6 months

Multivariate Cox regression results are shown in Table 4. The CT-PCI change ratio (ΔPCI), change in grade of ascites and therapy line were independent indicators that were associated with OS. In addition,  lower ΔPCI value was correlated with longer OS (Fig. 2). The stable and reducing volume of grade of ascites after therapy was correlated with longer OS in patients with GCPM. The first-line therapy was associated with longer OS.

**Table 4:** Multivariate cox regression results of parameters obtained from enhanced CT for predicting long-term survival

Parameters	*B*	HR (95% CI)	*P*
Δ PCI	1.215	3.37 (1.873–6.062)	< 0.001
ascites grade change	1.267	3.550 (1.776–7.096)	< 0.001
Therapy line	0.486	1.626 (1.021–2.588)	0.04

*B* regression coefficient, *CI* confidence interval, *HR* hazard ratio, *PCI* peritoneal cancer index, *Δ PCI* PCI change ratio based on CT after treatment

### The performance of CT-PCI change compared with RECIST 1.1

**Fig. 3:**
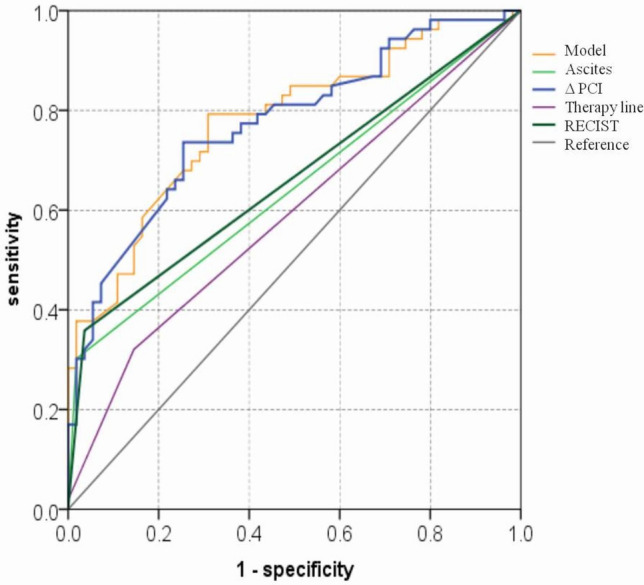
Receiver operating characteristic (ROC) curve analysis of CT analysis for predicting 12-month survival. The AUCs of ΔPCI (blue line), Ascites grade change (green line), therapy line (purple line), Mode (orange line) and RECIST 1.1 (dark green line) for predicting 12-month survival were 0.773, 0.642, 0.589, 0.778 and 0.661, respectively. A combined model was established using the following formula: Probability value= 1.215* ΔPCI + 1.267* ascites grade change + 0.486* therapy lines. Regarding to ascites grade change, reducing or stable classification was assigned as 0 and increasing classification was assigned as 1. First line therapy was assigned as 1 and second- or later- line therapy was assigned as 2

The performance of the CT-PCI change ratio (ΔPCI) with a cut-off value of greater than − 15%, therapy line with second- or later-line chemotherapy and ascites grade change after therapy with increasing grade to predict the high-risk group is summarized in Table 5 and Fig. 3. Based on the Cox regression result, the ΔPCI, ascites grade change, and therapy line were applied to construct a diagnostic model to predict 12-month OS in patients with GCPM.

**Table 5:** Performance of CT parameters and clinical characteristics to predict 12-month survival

Parameters	AUC (95% CI)	Cut-off	Sen (%)	Spe (%)	PPV (%)	NPV (%)	ACU (%)
Δ PCI	0.773 (0.685–0.861)	≥ − 15%	70.2	74.5	74.1	70.7	73.2
Ascites grade change	0.642 (0.537–0.747)	Increasing	29.8	98.2	94.4	57.4	63.4
Therapy line	0.589 (0.481–0.697)	Second- or later- line	31.6	85.5	69.2	54.7	58
Model	0.778 (0.690–0.865)	≥ 0.3	77.2	69.1	72.1	74.5	73.2

*AUC* area under curve, *ACU* accuracy, *PCI* peritoneal cancer index, *NPV* negative predictive value, *PPV* positive predictive value, *Δ PCI* change ratio of CT-PCI after therapy, *Sen* sensitivity, *Spe* specificity

**Fig. 4:**
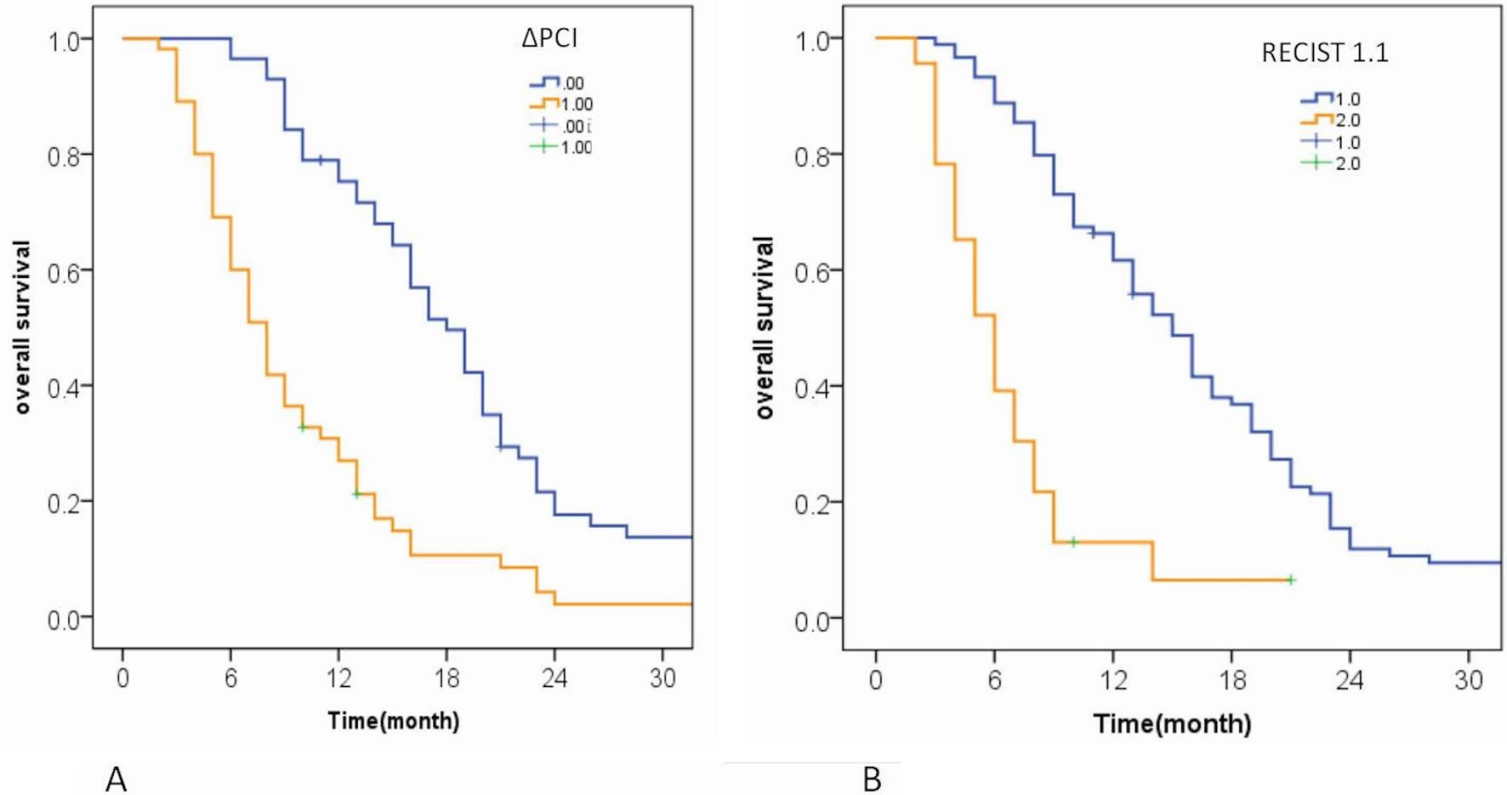
Kaplan‒Meier curves for overall survival of patients with PM in gastric cancer in the high-risk group and low-risk group based on ΔPCI (**A**) and RECIST 1.1 (**B**). Patients with ΔPCI less than −15% (blue line, low-risk group) showed a significant difference in OS compared to patients with ΔPCI greater than −15% (orange line, high-risk group) in **A**. Patients diagnosed as non-PD (blue line, low- risk group) got longer OS than in patients with PD (orange line, high-risk group) in **B**. Curves were compared using the log-rank test. *ΔPCI*, the PCI change ratio based on CT after treatment; *PD*, progressive disease

**Fig. 5:**
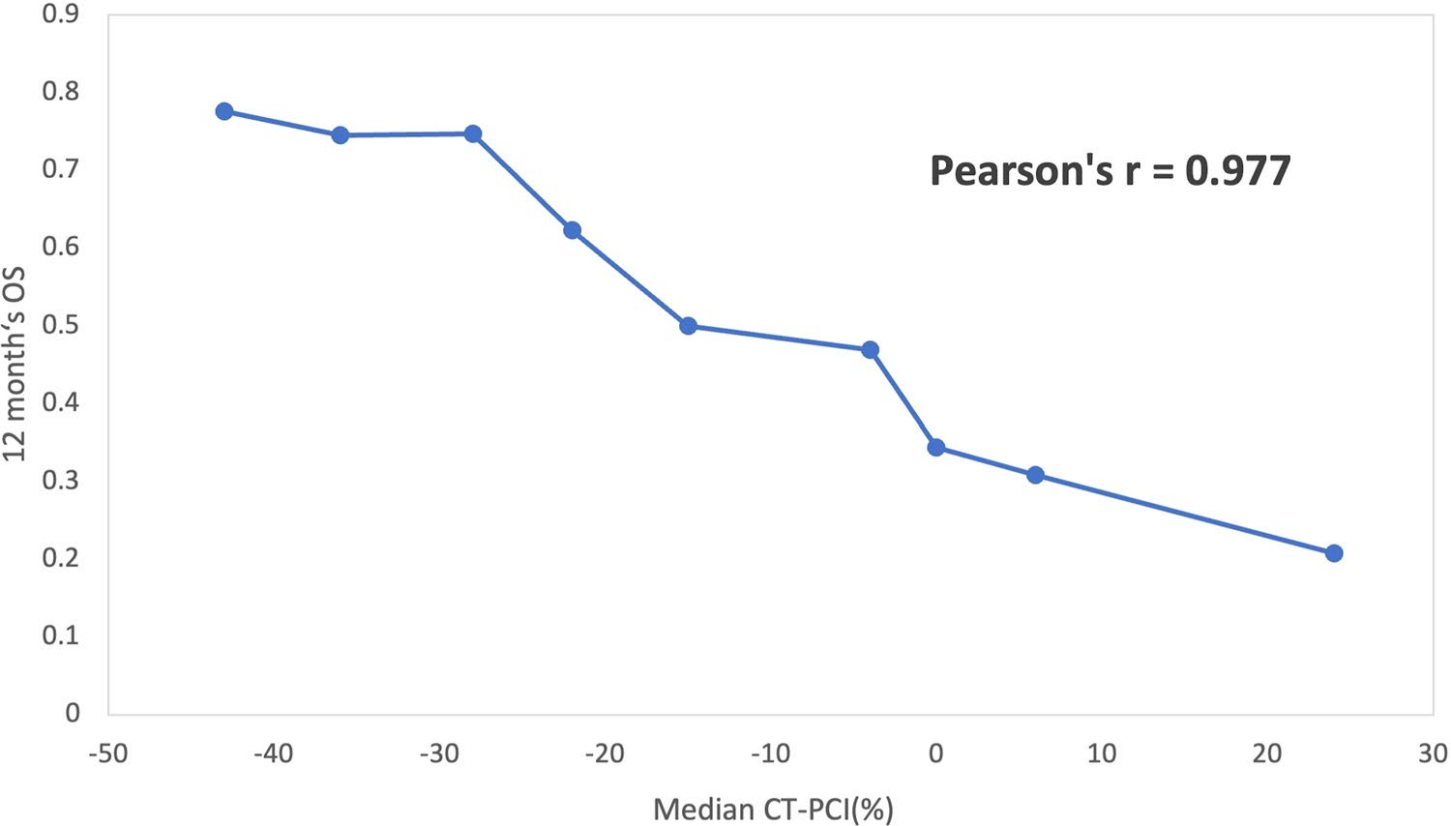
Subpopulation treatment effect pattern plot (STEPP) analysis of 12-month OS across ΔPCI. *ΔPCI * was the PCI change ratio based on CT after treatment

This model produced a remarkable diagnostic ability with an AUC of 0.778 comparable to ΔPCI (AUC = 0.773). Therefore, ΔPCI was chosen for comparison with RECIST guidelines to predict the prognosis. To predict 12-month OS of patients with GCPM, ΔPCI with AUC of 0.773 was significantly higher than RECIST guidelines with AUC of 0.661 (*P* = 0.026) (Fig. 4). The results of STEPP analysis are shown in Fig. 5, the Pearson’s r between the median change ratio of CT-PCI and 12-month OS was − 0.977, indicating that the CT-PCI change ratio was a good and valuable prognostic factor.

### Clinical usefulness

**Fig. 6:**
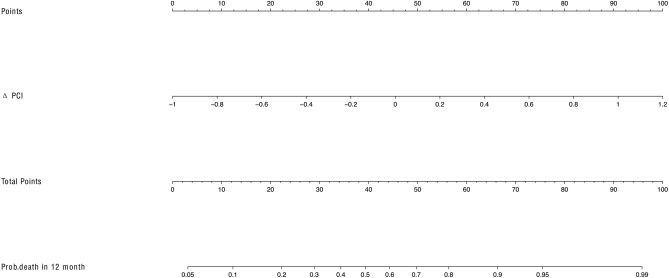
Nomogram developed with the ΔPCI in patients with peritoneal metastasis in gastric cancer. *Prob* survival was probability of survival. *ΔPCI* was CT-PCI change ratio after therapy

**Fig. 7:**
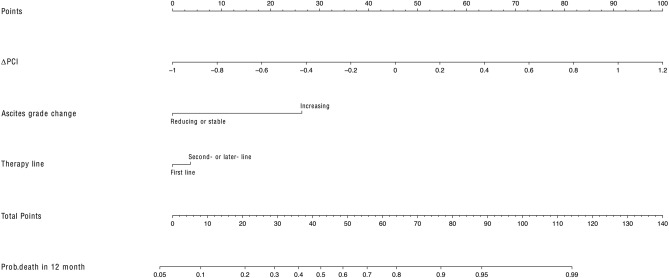
Nomogram developed with a model containing ΔPCI, ascites grade change and therapy line in patients with peritoneal metastasis in gastric cancer. *Prob survival* was probability of survival. *ΔPCI* was CT-PCI change ratio after therapy

To enhance clinical utility, two nomograms were established to predict overall survival for patients with peritoneal metastasis in gastric cancer, one solely based on ΔPCI and the other incorporating the comprehensive model (including ΔPCI, ascites grade change, and therapy line) (Figs. 6, 7). These nomograms could predict the probability of death within 12 months, with a range from 0 to 1. A probability closer to 1 indicated a higher likelihood of mortality. Patients diagnosed with peritoneal metastasis in gastric cancer could benefit from this predictive model.

## Discussion

The emerging comprehensive treatment strategy improved survival in patients with PM from gastrointestinal cancer (Verwaal et al. 2003). The assessment of PM in gastric cancer after chemotherapy is crucial to allow planning the next treatment strategy. Enhanced abdominal and pelvic CT is routinely used after two or three treatment cycles to evaluate the efficacy of the therapy. If PM is assessed as stable disease or regression, the regimen will be continuously administered to patients until the disease progresses or the toxicity is beyond tolerance. After the disease progresses, the choice of second-line or follow-up treatment depends on previous treatment and physical state. Timely measurement of treatment response using imaging modalities is essential.

Currently, CT and staging laparoscopy are the two most common modalities to detect peritoneal metastases (Healy 2001; Saiz Martínez et al. 2021; Yan et al. 2008; Laghi et al. 2017). The main purpose of laparoscopy is to detect occult peritoneal disease that cannot be definitively diagnosed by imaging examinations and to determine the correct therapeutic strategy for the treatment of advanced gastric cancer (Fukagawa 2019; Coburn et al. 2017). The indication for laparoscopy is in patients with equivocal CT findings and repeated laparoscopy is important after chemotherapy, especially to allow decision making on the need for conversion surgery (Fukagawa 2019). However, according to the 6th edition of the Japanese Gastric Cancer Treatment Guidelines, laparoscopy is weakly recommended to establish the therapeutic strategy for patients with advanced GC with extensive PM (Japanese Gastric Cancer Association 2023). Thus, for unresectable gastric cancer with PM and if there was convincing evidence of widespread visceral metastatic or PM on imaging and the patient was not deemed medically fit for curative surgery, laparoscopy was not required. Patients in this study were clinical stage IV with definite peritoneal metastasis diagnosed by CT, which was unsuitable for laparoscopic exploration. The accuracy in evaluating the tumor burden of peritoneal metastases relies on the identification of lesions on CT. In Sugarbaker’s research, the false negative percentage ranges from 10 to 28% when the implant size is greater than 0.5 cm (Jacquet et al. 1993; Sugarbaker 2018). Low et al. reported that CT predicted 81% (13/16) of patients with moderate (PCI 10–20) to larger (PCI > 20) volume peritoneal tumor in appendiceal and ovarian cancer (Low et al. 2015). In the study by Sartor et al., the increase in CT-PCI was proved to be statistically significantly associated with advanced clinical stage of ovarian cancer and higher CT-PCI was significantly associated with poor ovarian cancer-specific survival (Sartor et al. 2020). They believe that patient outcomes are particularly interesting to highlight and are likely more relevant than surgical findings as a standard of reference for imaging studies. Based on the results of previous studies, we believe that CT evaluation is feasible under high PCI conditions. Our study also proved that the high CT-PCI value and the increasing CT-PCI were the high risk of poor survival in gastric cancer patients with peritoneal metastases.

PCI strictly related to the extent and distribution of PM emerged as a prognostical significant predictor of survival outcomes for most types of PM (Horvath et al. 2018). PCI has been used to assess the tumor burden in PM in previous reports. Previous studies demonstrated that PCI based on CT is a feasible and reproducible modality that presented a better correlation with PM surgery. CT-PCI correlated well with surgical PCI, and CT-PCI could predict the complete cytoreduction of PM (Panagiotopoulou et al. 2021; Laghi et al. 2017; Flicek et al. 2016). However, little is known about the use of the change in PCI based on CT after therapy compared to that of baseline CT. This study is the first to analyze the change in PCI between baseline CT and CT after therapy to predict the prognosis. We found that ΔPCI could be utilized as a response criterion to predict PM OS in gastric cancer patients treated with chemotherapy. Size-based criteria, such as RECIST criteria, were limited in evaluating the response of PM treatment. By comparing RECIST criteria, CT-PCI that includes 13 regions of the abdomen and small intestine could better assess tumor burden. Using Sugarbaker’s PCI score system, CT-PCI based on the distribution and size of PM in gastric cancer can quantitatively assess these lesions that are unsuited for size-based criteria. Therefore, our results also found that the AUC of ΔPCI was significantly higher than that of the RECIST guidelines in predicting OS in patients with PM. Reliable prognostic indicators may suggest that clinicians choose appropriate management options for patients with treatment resistance.

In this study, change in ascites grade and therapy line were also independent indicators for predicting OS. The reduced and stable volume of ascites was related to longer OS. The PM in gastric cancer may increase capillary permeability and fluid production or decrease absorption and produce abdominal ascites (Diop et al. 2014). Honda et al. found that the grade of ascites prior to treatment could better predict the prognosis of patients with gastric cancer (Honda et al. 2020). The trends in ascites after chemotherapy helped decide to change a regimen to a later line. First-line therapy was also positively associated with OS by previous reports. This was also revealed in the phase III study by Koizumi et al. that first-line treatment significantly prolonged median OS by 13.0 months and proved better efficacy in patients with peritoneal metastasis (Koizumi et al. 2008). However, patients who failed first-line treatment gained a median OS of only 7.7 months (Nishina et al. 2016).

In addition, data from previous literature had reported that the presence of extraperitoneal metastasis was the independent prognostic risk factor for patients with GCPM (Chen et al. 2018). In our supplemental study, an analysis was conducted in patients without liver metastases and/or bone metastases. A model was established with change ratio of CT-PCI, change of ascites grade, and line of therapy based on the multivariate Cox regression results to predict 12 months of death. The analysis suggested that it had no great effect when excluding patients with liver metastases and/or bone metastases.

The current investigation had several limitations. First, PM in gastric cancer was not pathologically confirmed point-by-point; verification by biopsy was not routinely performed in clinical practice. Second, the retrospective study was carried out in a single center; further prospective research is required from multiple centers to validate our results. Third, the treatment regimen for PM was not standardized, which may have caused variations in the present study results. Fourth, the diagnosis of PM lesions less than 5 mm is challenging on CT, and these lesions may be underestimated in our study. This is a common diagnostic dilemma even with PET-CT, and the bias result produced by subjectively assessing a PM lesion less than 5 mm can be reduced by assessing lesion-by-lesion via consecutive CT examinations. Fifth, 38.4% of patients underwent resection of gastric cancer; the scar due to surgery may have been misdiagnosed as metastasis, which may have yielded bias in our results.

## Conclusion

In conclusion, the result of our study showed that dynamic change in CT-PCI after chemotherapy proved excellent performance in predicting overall survival, especially in the high PCI population of GCPM, including patients with unresectable primary GC with synchronic or metachronous PM and those with metachronous PM after gastrectomy during follow-up. The change ratio of PCI after chemotherapy can be a valuable prognostic indicator and help clinicians choose appropriate treatment strategies.

## Data Availability

The data that support the findings of this study are available from the corresponding author upon reasonable request.

## Abbreviations

PCI: Peritoneal cancer index
ΔPCI: PCI change ratio based on CT after treatment
OS: Overall survival
CR: Complete disease
PR: Partial response
SD: Stable disease
PD: Progressive disease
